# External mechanical perturbations challenge postural stability in dogs

**DOI:** 10.3389/fvets.2023.1249951

**Published:** 2023-09-18

**Authors:** Christiane Lutonsky, Christian Peham, Marion Mucha, Bianca Reicher, Rita Gaspar, Alexander Tichy, Barbara Bockstahler

**Affiliations:** ^1^Department of Companion Animals and Horses, University Clinic for Small Animals, Small Animal Surgery, Section of Physical Therapy, University of Veterinary Medicine, Vienna, Austria; ^2^Department of Companion Animals and Horses, University Clinic for Horses, Movement Science Group, University of Veterinary Medicine, Vienna, Austria; ^3^Department of Biomedical Sciences, Bioinformatics and Biostatistics Platform, University of Veterinary Medicine, Vienna, Austria

**Keywords:** canine balance, center of pressure, postural stability, posturography, veterinary rehabilitation, external mechanical perturbations

## Abstract

This study aimed to explore the effect of external mechanical perturbations on postural stability (PS) in dogs using the body center of pressure (COP). Thirteen sound adult dogs were included in this study. PS was tested during quiet standing on a pressure measurement plate. The conditions included a standard standing measurement and external mechanical perturbations conducted using six settings on a motorized training platform with different intensities of speed and amplitude. Measurement conditions were compared using linear mixed-effects models, followed by multiple comparisons using Sidak’s alpha correction procedure. Compared with the standing measurement, external mechanical perturbations resulted in a significant increase in almost all COP parameters, indicating a challenge for the PS. Furthermore, an increase in amplitude had a greater effect than an increase in speed, whereas the combination of the highest intensities of amplitude and speed was not well tolerated by the dogs. The craniocaudal COP displacement was significantly greater than the mediolateral COP displacement during standing measurement and conditions with a small amplitude, whereas no significant difference was observed during settings with an increased amplitude. To the best of our knowledge, this is the first study to demonstrate the effects of a balance training device in dogs. Therefore, the intensity of the training programs on motorized platforms or similar devices can be controlled by the wobbling amplitude of the platform.

## 1. Introduction

Postural stability (PS) is the act of maintaining, achieving, or restoring balance during a posture or activity (1). This is achieved through interactions between the visual, somatosensory, and vestibular systems of the central nervous system (2). Body stabilization results from the transmission of information from the central nervous system to the musculoskeletal system (1).

A typical parameter used to evaluate PS in human patients is the center of pressure (COP) (3–9), which has recently been used in veterinary medicine (10–31). The COP is the location of the instantaneous vector of ground reaction forces and displays the trajectory of the center of mass of the body. During the ground contact, the position of the center of mass, and therefore the COP, changes continuously, resulting in a COP path (9). The COP moves within the base of support of the body. Balance is maintained by rapid COP movements to maintain the center of mass within the base of support (32, 33). If the COP exceeds the base of support, a protective step is taken to prevent falls (34). Therefore, the ability to restore balance is directly influenced by the position of the COP prior to perturbation (35). The COP can be measured easily using force and pressure measurement plates (10–20).

Previous evaluations in veterinary medicine were performed during static posturography, that is, during quiet standing with (11–16, 22, 24, 31) and without challenging PS (23), and during gait analysis in normal locomotion (10, 20, 21) and different tasks (17, 18). Moreover, the COP of the body (11, 14–16, 22, 23, 31) and that within the paws (10, 12, 13, 17–20) can be measured. Some measurements are conducted with the animal standing on a plate with all feet to calculate the body COP (11, 16, 22, 23, 31), whereas others evaluate it only between the forelimbs (11, 14, 15, 31) or hindlimbs (11, 31). The COP parameters are calculated based on the COP path. During posturography, the following body COP parameters are commonly described: The displacement of the COP in mediolateral (COP-MedLat) and craniocaudal (COP-CranCaud) directions; the support surface, which is the area determined by an ellipse that contains 90% of the points of the COP trajectory; (11) the COP speed; the statokinesiogram length; and length in the function of surface. The statokinesiogram length is the length of the line joining the points of the COP trajectory. It is a measure of the effort needed to maintain an upright position and therefore measures the efficacy of the postural system (36). The length in the function of surface is the correlation between the COP length and its surface. It provides information on the accuracy of the PS and the effort made by the subject to maintain an upright position (15, 37). An increase in the COP parameters is associated with impaired PS (16, 22, 23).

During standard standing measurement, veterinary research has focused on the body COP of sound (28, 31) and lame (15) horses, foals (22), senile (16, 24) and lame (11, 14) dogs, and the COP within the paws of lame dogs (12, 13). In horses, the COP-MedLat was significantly larger than the COP-CranCaud during standing measurement of the forelimbs. Based on suggestions in human medicine, Pitti et al. (15) proposed that the larger craniocaudal diameter of the base of support in horses is responsible for the more profound stability in the craniocaudal direction. While similar comparisons have not yet been performed in dogs, it has been proposed that the support surface has a wider diameter in the mediolateral direction than that in the craniocaudal direction during measurements between the fore (11, 14) and hind limbs (11). Up to this point, according to the authors’ knowledge, there have been no investigations into the influence of different body conformations on the support surface. However, COP-parameters are significantly influenced by the weight, height, and length in dogs (16). Increased body COP displacement during static posturography is associated with the age and health status of the animals. In foals, (22), increases in the COP-MedLat and COP-CranCaud were attributed to a poorly developed PS. Similar patterns of COP parameters have been described in senile dogs. Again, these results are discussed as signs of decreased PS (16, 24). This finding is consistent with the results of previous human medical research. Children and elderly individuals show increased support surface and, therefore, decreased PS compared to healthy adults (38). Furthermore, the authors suggested that the significant differences between the senile and younger dogs could be the result of joint pain and other comorbidities associated with aging (16), as described in dogs with osteoarthrosis. Cubarthrosis and gonarthrosis result in a significant increase in COP-MedLat, COP-CranCaud, and support surface compared to a control group (11, 14). Similarly, human medical research has revealed a negative effect of experimentally induced pain on postural stability in healthy adults (39).

Dynamic balance tests challenge the standing postural stability during external perturbations or dynamic conditions, including mechanical, sensory, or combined stimuli (40). Although the loss of visual input in horses results in a significant increase in COP-MedLat, COP-CranCaud, and mediolateral COP velocity (23), dynamic balancing tests during the investigation of COP parameters have not yet been performed in canines. External mechanical perturbations, including different surfaces (41–43), waist pulls (32, 35), mechanical platforms (44–49) and narrowing of the base of support with a single-leg (6, 41, 50–52) and tandem stance (standing in a heel-to-toe position) (53–55), are commonly used in human medical research to challenge PS. Increased base of support by a wider stance resulted in improvements in balance performance, mainly in COP-MedLat in healthy adults (56), while the single-leg stance increased total COP-MedLat, COP-CranCaud, and mean COP-CranCaud (52). In patients with anterior cruciate ligament ruptures, the single-leg stance resulted in a significant increase in the total COP-MedLat compared with the control group (6). Furthermore, the functional base of support, defined as the area used to maintain balance, decreases during the aging process. Therefore, a decrease in functional base of support results in an impaired ability to maintain or restore balance (57, 58). Using a motorized platform, researchers found a significant increase during sinusoidal perturbations at an amplitude of 5° at a high frequency (0.50 Hz) compared to a low frequency (0.25 Hz) in statokinesiogram length, whereas the frequency did not have a significant effect on COP-MedLat and COP-CranCaud. However, a strong correlation between statokinesiogram length and COP-MedLat was observed under both conditions (48). Furthermore, control participants and patients with Parkinson’s disease were less challenged by anterior–posterior perturbations than that by lateral perturbations, and diseased patients showed a less efficient postural strategy (49).

As described above, many approaches to challenging PS have been described in humans, most of which cannot be applied to animals. Similar to the single-leg stance, three-legged standing tests are commonly used to assess strength and balance in dogs (59). However, this test is subject to high variability among practitioners (60), and standing measurement is difficult to perform over a sufficient period (16). Therefore, this test condition may lack validity and be difficult to perform in orthopedically and neurologically diseased animals. Dynamic tests, such as walking Figure 8’s and stepping over Cavaletti rail obstacles, have been proposed to test dynamic balance and spatial awareness with low variability among patients and practitioners (60). The latter led to significant differences in the COP parameters compared with normal walking (17). However, static posturography during external perturbations has not been addressed in the literature.

Mechanical platforms can be used to measure the PS in animals under challenging conditions. The difficulty can be adjusted to the animal’s fitness level by changing the speed and angulation to a horizontal plane (amplitude) of the upward and downward motions. These properties enable the evaluation of PS during repeatable measurements under fixed conditions.

Veterinary research should focus on establishing measurement procedures that assess the influence of external perturbations on the PS in sound and orthopedically or neurologically diseased animals. As a first step toward evaluating the effect of external perturbations on PS in dogs, this study aimed to assess the effect of external perturbations on COP parameters in sound dogs in different settings on a motorized training platform. In the future, this may serve as an extension of conventional measurements of ground reaction forces, which provide insight into limb loading but are not adequate to describe the sway of the whole-body.

We hypothesized that external mechanical perturbations challenge PS in dogs, which is reflected in the COP parameters of the body. Furthermore, higher amplitude and speed settings of the motorized platform result in signs of increased instability, and an increase in amplitude has a stronger effect than an increase in speed.

## 2. Materials and methods

### 2.1. Ethics statement

This study was approved by the Ethics and Animal Welfare Committee of the University of Veterinary Medicine, Vienna, in accordance with the University’s guidelines for “Good Scientific Practice” (ETK-131/09/2021).

### 2.2. Animals and inclusion criteria

The sample size was calculated using G*Power 3.1 based on the results of a pilot study (Ethics and Animal Welfare Committee of the University of Veterinary Medicine approval number ETK-101/06/2020), which resulted in a total of 10 dogs, assuming a power of 80% and a type I error probability of 5%.

Dogs with pre-existing orthopedic and neurological conditions or any other diseases that could negatively impact PS, such as diseases of the inner ear and reduced vision, were preliminarily excluded. Further inclusion criteria were ectomorph or mesomorph conformation, body weight of 15–35 kg, and age of 2–8 years. Additionally, gait analysis was performed using a pressure measurement plate (FDM Type 2; Zebris Medical GmbH, Allgäu, Germany) to obtain an objective lameness assessment (section 2.3.1). The symmetry indices (SI) of peak vertical force (PFz) and vertical impulse (IFz) had to be below 3%, which is a margin that has been repeatedly used to distinguish between sound and lame dogs (10, 61, 62).

Fifteen dogs were initially included in the study. Two dogs were excluded due to their SI for PFz and/or IFz > 3%. The remaining study population included seven female and six male animals (four mixed breeds, four Border Collies, one Australian Shepherd, two Labrador Retrievers, one Belgian Malinois, and one Belgian Laekenois). The mean age and body mass were 4.29 ± 2.15 years (median = 4.33, minimum = 2.00, maximum = 7.83), and 22.18 ± 4.14 kg (median = 21.50, minimum = 16.70, maximum = 29.00), respectively.

### 2.3. Procedure and equipment

#### 2.3.1. Initial examination

A 203 × 54.2 cm pressure measurement plate (FDM Type 2, Zebris Medical GmbH, Allgäu, Germany) was used during the initial examination for objective gait analysis. The plate was covered with a black, 1-mm thick rubber mat composed of polyvinyl chloride to avoid slipping.

First, the dogs were allowed to move freely to acclimate to the measurement room. The measurements were performed during walking and trotting until at least five valid passes were collected for each gait. A valid pass was defined as a walk or trot in which the dog crossed the plate without changing pace, turning its head, pulling on the leash, or touching the owner. The difference in speed at which the dogs crossed the plate had to be within a range of ±0.3 m/s and an acceleration of ±0.5 m/s^2^ (63–65).

#### 2.3.2. Posturography

**Figure 1:**
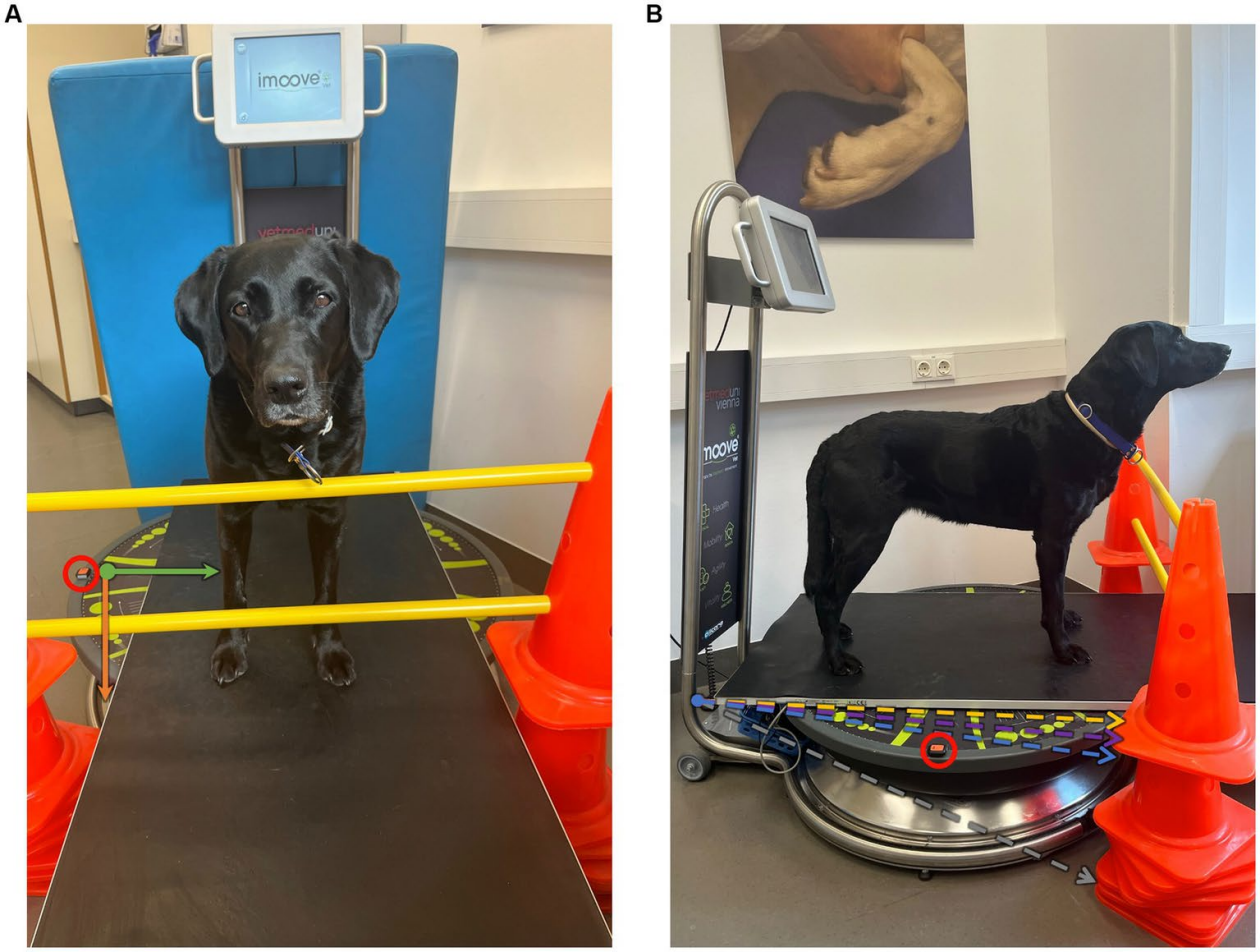
Experimental setup: **(A)** frontal and **(B)** lateral views showing the motorized platform in the neutral position, which is used for the standard standing measurement. The accelerometer is placed on the right side of the motorized platform (within the red circle). The movement of the platform is measured on the X- (green), Y- (orange), and Z-axes using Xsens DOT sensor **(A)**. The amplitudes are illustrated in dotted lines to a horizontal plane **(B)**, including 8° in gray (maximal amplitude of the device); 0.8° in yellow (used during Speed-20%, Speed-30%); 1.6° in purple (Amplitude-20%, Combination-20%); and 2.4° (Amplitude-30%, Combination-30%).

Static measurements were conducted using a 149 × 54.2 cm pressure measurement plate (FDM-1.5, Zebris Medical GmbH, Allgäu, Germany), which was placed on a motorized platform (Imoove-vet® platform, Allcare Innovations, 26500 Bourg les Valence, France). This platform uses Elispheric® movement that results from a combination of 3 movements (rotation, eccentricity, and inclination) giving an elliptical or spiral outline in 3 dimensions (66). The pressure measurement plate measured the pressure on the dog’s paws using 15,360 piezoelectric sensors at a sampling rate of 100 Hz. The plate was covered with a black, 1-mm-thick rubber mat made of polyvinyl chloride. As the plate was longer than the standing surface of the platform, two cavaletti were used to ensure that the dogs did not pass over the edge. Each measurement run was filmed with a camera (Panasonic model NV-MX500) to evaluate head, limb, and tail movements. The movement of the platform during the measurement conditions was measured on the X-, Y-, and Z-axes using an accelerometer (Xsens DOT sensor) placed on the right side of the standing surface (Figure 1).

The owner led the dog onto the pressure measurement plate and halted it in a straight and square position. During data collection, the owner stood close to the dog without physical contact, to discourage movement. All conditions were measured for 1 min and repeated three times. To avoid the effect of fatigue on the data, each dog was tested on three separate days (two conditions per day), with at least 2 days in between. A short break of 1 min was scheduled after each measurement.

First, standard standing measurements (11–14) were conducted in the neutral position of the motorized platform (Figure 1) on all measurement days to accustom the dog to the situation and practice quiet standing. Subsequently, testing conditions with different settings were performed randomly on a motorized platform. The platform allowed a maximum angulation of 8° (100% of amplitude, Figure 1) and maximum speed of 1 Hz or 60 rounds per min (rpm; 100% of speed) (66). The settings used in this study are listed in Table 1. These included settings of increased speed and fixed amplitude (Speed-20%, Speed-30%), increased amplitude and fixed speed (Amplitude-20%, Amplitude-30%), and a combination of speed and amplitude (Combination-20%, Combination-30%). If an animal showed excessive paw or head movement during a condition, the measurement was discontinued and labeled as not well tolerated by the dog.

**Table 1:** Overview of the measurement conditions.

Setting	Speed	Amplitude	Effect of
Speed-20%	20%/12 rpm	10%/0.8°	Speed
Speed-30%	30%/18 rpm	10%/0.8°	
Amplitude-20%	10%/6 rpm	20%/1.6°	Amplitude
Amplitude-30%	10%/6 rpm	30%/2.4°	
Combination-20%	20%/12 rpm	20%/1.6°	Combined settings
Combination-30%	30%/18 rpm	30%/2.4°	

The speed in percentage of total speed (%) and rpm and amplitude in percentage (%) of total amplitude and degrees (°).

**Figure 2:**
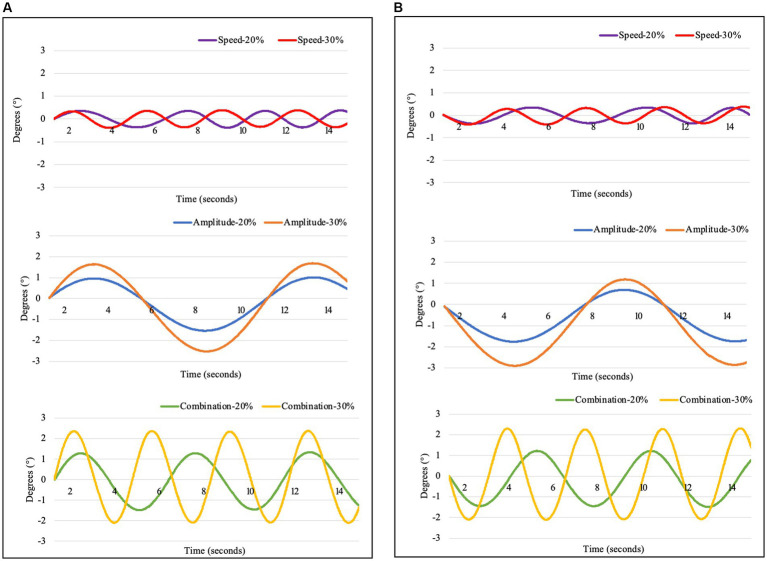
Movement of the platform, measured with an Xsens DOT sensor in **(A)** laterolateral (X-axis) and **(B)** craniocaudal (Y-axis) directions during the investigated settings including Speed-20% (purple): Speed 20%, Amplitude 10%; Speed-30% (red): Speed 30%, Amplitude 10%; Amplitude-20% (blue): Speed 10%, Amplitude 20%; Amplitude-30% (orange): Speed 10%, Amplitude 30%; Combination-20% (green): Speed 20%, Amplitude 20%; and Combination-30% (yellow): Speed 30%, Amplitude 30%.

The motorized platform showed a sinusoidal motion consistent in the craniocaudal and laterolateral directions (Figure 2).

### 2.4. Data analysis

A custom software Pressure Analyzer (Michael Schwanda, version 4.8.5.0) was used for data analysis, which was then exported to Microsoft Excel 2016. Pawprints were manually assigned to the corresponding limbs. Out of each 1-min measurement, a valid period of 20 s, indicating no movements of the head or paws and only minimal tail movements was selected. If a continuous valid period was not achieved, head and paw movements were manually cropped. A measurement was considered insufficient if a valid period of less than 20 s was selected. To assess the different settings, the three-dimensional movements in all directions were included in the selected timeframes. If this was not possible, the measurements were excluded from the data analysis.

### 2.5. Parameters under investigation

The following parameters were used for the evaluation of the inclusion criteria during the lameness assessment in walk and trot using the pressure measurement plate:

- The mean speed (m/s) and acceleration (m/s^2^) were calculated for the left forelimb based on subsequent steps.
- Symmetry index (SI) expressed as a percentage (SI%), was calculated for both parameters (PFz and IFz) according to the following equation:
SIXFz(%)=abs([XFzLLx−XFzRLx]/[XFzLLx+XFzRLx])∗100

where XFz is the mean value of peak vertical force (PFz) or vertical impulse (IFz) of valid steps, LLx is the left front or hindlimb, and RLx is the right front or hind; Perfect symmetry between the right and left front or hindlimbs was assigned a value of 0%.

Using the body COP, the following parameters were assessed:

- COP-MedLat: Mean deviation on the lateral axis (mm) measures alterations in the center of mass load distribution on the sagittal axis, and smaller displacement is associated with better stability (16, 24).
- COP-CranCaud: Mean deviation on the craniocaudal axis (mm); a smaller displacement is associated with better stability (16, 24).
- Support surface (mm^2^): or statokinesiogram, the area determined by an ellipse that contains 90% of the points of the COP trajectory, gauges the changes in orientation of a standing subject; smaller displacement is associated with better stability (11).
- Statokinesiogram length (path length, m): the length of the line that joins the points of the COP trajectory, a measure of the effort needed to maintain an upright station; and a parameter linked to support surface, which measures the efficiency of the postural system (36). In other words, if support surface is equal, a lower statokinesiogram length indicates a smaller expenditure of energy and, hence, a more efficient PS, and a higher value indicates more instability (14, 15).
- Length as a function of surface: Correlation between COP length and its surface. This provides information on the accuracy of the PS and the effort made by the subject; a higher value indicates greater instability (15, 37).
$$\begin{array}{l} \mathrm{Length}\;\mathrm{as}\;a\;\mathrm{function of surface}=\mathrm{support surface}\\ \;\;\;\;\;\;\;\;\;\;\;\;\;\;\;\;\;\;\;\;\;\;\;\;\;\;\;\;\;\;\;\;\;\;\;\;\;\;\;\;\;\;\;\;\;\;\;\;\;\;\;\;\;\;\;\;\;\;\;\;\;\;\;\;\;\;\;\;\;\;\;\;\;\;/\mathrm{statokinesiogram length} \end{array}$$
- Mean speed (mm/s) of COP sway (COP-Speed).

### 2.6. Statistical analysis

All statistical analyses were performed using IBM SPSS v27. The effects of different measurement conditions on the parameters were analyzed using linear mixed-effects models in which the conditions were added as fixed factors to the model. Sidak’s alpha correction was applied for multiple comparisons. The assumption of a normal distribution was tested using the Shapiro–Wilk test. For all analyses, a value of *p* <5% (*p* < 0.05) was observed as significant.

## 3. Results

### 3.1. Symmetry index

The SI values for PFz and IFz during walking and trotting are listed in Table 2. All dogs included in this study had a SI of PFz and IFz < 3% during walking and trotting.

**Table 2:** Symmetry index (SI) of peak vertical force (PFz) and vertical impulse (IFz) during the initial examination in walk and trot.

	SI PFz (%)		SI IFz (%)	
	Mean ± SD			
	Walk	Trot	Walk	Trot
Forelimbs	0.67 ± 0.37	0.77 ± 0.79	1.13 ± 0.79	1.33 ± 0.93
Hindlimbs	1.08 ± 0.78	1.21 ± 0.62	1.54 ± 0.65	0.89 ± 0.66

### 3.2. Valid measurements

All dogs tolerated conditions Speed-20%, Speed-30%, and Combination-20%; therefore, the data analysis included measurements from 13 dogs. The amplitude settings (Amplitude-20%, Amplitude-30%) were not tolerated by one dog, and condition Combination-30% led to valid measurements in 9 out of 13 dogs.

### 3.3. Center of pressure

**Figure 3:**
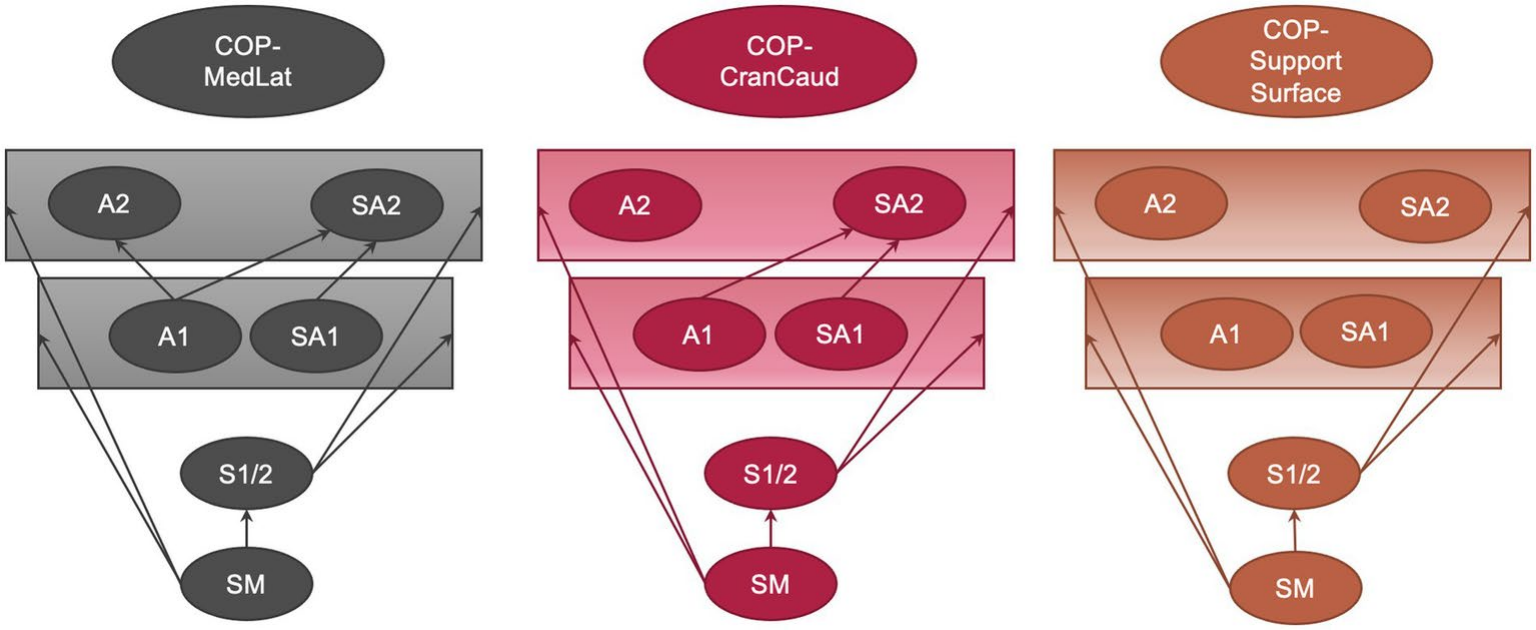
Group comparison of the mediolateral (COP-MedLat) and craniocaudal (COP-CranCaud) COP-displacement and the support surface between all measured conditions including standard standing measurement, speed settings (Speed-20%: speed 20%, amplitude 10%; Speed-30%: speed 30%, amplitude 10%); amplitude settings (Amplitude-20%: speed 10%, amplitude 20%; Amplitude-30%: speed 10%, amplitude 30%); and combined setting (Combination-20%: speed 20%, amplitude 20%; Combination-30%: speed 30%, amplitude 30%). The arrows indicate significant differences between the conditions (*p* < 0.05).

The main results are shown in Figure 3. Compared with standing measurement, all tested conditions led to a significant increase in COP-MedLat, COP-CranCaud, support surface, and length in the function of surface, except for Combination-30% (just out of significance). No significant differences were found between statokinesiogram length and COP-Speed, except for Combination-30%. The mean values, standard deviations, and upper and lower limits of the 95% confidence interval can be found in Table 3, all *p*-values of the group comparisons are summarized in Table 4.

**Table 3:** Mean values, standard deviation (SD), lower (LL) and upper (UL) limits of the 95% confidence interval (CI) of the conditions standing measurement, speed settings (Speed-20%, Speed-30%), amplitude settings (Amplitude-20%, Amplitude-30%), and combined settings (Combination-20%, Combination-30%) for mediolateral COP-displacement (COP-MedLat), craniocaudal COP-displacement (COP-CranCaud), support surface, statokinesiogram length, length in the function of surface and COP speed.

Condition		Standing measurement	Speed-20%	Speed-30%	Amplitude-20%	Amplitude-30%	Combination-20%	Combination-30%
**COP-MedLat (mm)**								
Mean value ± SD		5.94 ± 0.32^1,2,3,4,5,6,§^	23.23 ± 0.83^*,3,4,5,6,§^	25.42 ± 1.24^*,3,4,5,6,§^	39.38 ± 1.73^*1,2,5,6^	51.24 ± 2.17^*1,2,3^	41.15 ± 2.13^*1,2,(4),6^	58.77 ± 3.33^*1,2,3,4^
95% CI	LL	5.25	21.42	22.71	35.58	46.46	36.51	51.09
	UL	6.63	25.04	28.12	43.18	56.02	45.79	66.45
**COP-CranCaud (mm)**								
Mean value ± SD		12.55 ± 0.65^1,2,3,4,5,6,§^	30.26 ± 1.06^*,3,4,5,6,§^	32.24 ± 1.57^*,3,4,5,6,§^	43.85 ± 2.09^*1,2^	50.81 ± 1.67^*1,2^	46.22 ± 2.58^*1,2^	66.92 ± 4.24^*1,2^
95% CI	LL	11.14	27.95	28.82	39.26	47.13	40.61	57.15
	UL	13.97	32.57	35.66	48.45	54.49	51.84	76.69
**Support surface (mm**^**2**^**)**								
Mean value ± SD		35.73 ± 4.21^1,2,3,4,5,6^	566.06 ± 52.30^*,3,4,5,6^	701.16 ± 82.59^*,3,4,5,6^	1813.90 ± 196.00^*1,2,(6)^	2836.70 ± 255.31^*1,2^	2062.62 ± 268.25^*1,2^	4044.66 ± 534.26^*1,2^
95% CI	LL	26.57	452.11	521.22	1382.51	2274.77	1478.15	2812.66
	UL	44.89	680.01	881.09	2245.29	3398.63	2647.09	5276.67
**Statokinesiogram length (m)**								
Mean value ± SD		1.08 ± 0.12	1.63 ± 0.24	1.50 ± 0.26	1.64 ± 0.24	1.53 ± 0.24	1.24 ± 0.24	1.49 ± 0.33
95% CI	LL	0.81	1.11	0.93	1.12	1.00	0.73	0.74
	UL	1.35	2.16	2.08	2.17	2.07	1.76	2.25
**Length in the function of surface**								
Mean value ± SD		0.05 ± 0.01^1,2,3,4,5,(6)^	0.53 ± 0.11^*,4,5^	0.77 ± 0.13^*,4,(5)^	1.41 ± 0.24^*^	2.33 ± 0.29^*1,2^	2.54 ± 0.47^*1,(2)^	4.63 ± 1.10 ^(*)^
95% CI	LL	0.03	0.29	0.48	0.89	1.69	1.52	2.09
	UL	0.07	0.77	1.05	1.93	2.97	3.57	7.17
**COP-Speed (mm/s)**								
Mean value ± SD		116.45 ± 3.82^6^	127.87 ± 5.61	136.76 ± 6.38	135.48 ± 6.80	132.09 ± 5.80	133.21 ± 5.56	147.92 ± 6.37^*^
95% CI	LL	108.13	115.66	122.87	120.51	119.31	121.11	133.22
	UL	124.77	140.09	150.65	150.44	144.86	145.32	162.62

^*^Significant difference between standard standing measurement and the remaining conditions (*p* < 0.05), ^1^significant difference to Speed-20%, ^2^significant differences to Speed-30%, ^3^significant differences to Amplitude-20%, ^4^significant differences to Amplitude-30%, ^5^significant difference to Combination-20%, ^6^significant difference to Combination-30%, ^§^ significant differences between COP-MedLat and COP-CranCaud under the same conditions. ^()^*p* ≤ 0.065.

**Table 4:** *P*-values of the group comparisons of the conditions standing measurement, speed settings (Speed-20%, Speed-30%), amplitude settings (Amplitude-20%, Amplitude-30%), and combined settings (Combination-20%, Combination-30%) for mediolateral COP-displacement (COP-MedLat), craniocaudal COP-displacement (COP-CranCaud), support surface, Statokinesiogram Length, Length in the function of surface and COP speed.

Condition (I)	Condition (J)	*p-values*					
		COP-MedLat	COP-CranCaud	Support surface	Statokinesiogram length	Length in the function of surface	COP-Speed
Standing measurement	Speed-20%	<0.001^*^	<0.001^*^	<0.001^*^	0.699	0.020^*^	0.907
	Speed-30%	<0.001^*^	<0.001^*^	<0.001^*^	0.974	0.003^*^	0.239
	Amplitude-20%	<0.001^*^	<0.001^*^	<0.001^*^	0.674	0.003^*^	0.421
	Amplitude-30%	<0.001^*^	<0.001^*^	<0.001^*^	0.925	0.000^*^	0.540
	Combination-20%	<0.001^*^	<0.001^*^	<0.001^*^	1.000	0.004^*^	0.364
	Combination-30%	<0.001^*^	<0.001^*^	0.001^*^	0.998	0.064^(*)^	0.018^*^
Speed-20%	SM	<0.001^*^	<0.001^*^	<0.001^*^	0.699	0.020^*^	0.907
	Speed-30%	0.973	1.000	0.985	1.000	0.986	1.000
	Amplitude-20%	<0.001^*^	0.001	0.001^*^	1.000	0.076	1.000
	Amplitude-30%	<0.001^*^	<0.001^*^	<0.001^*^	1.000	0.001^*^	1.000
	Combination-20%	<0.001^*^	0.001^*^	0.002^*^	0.998	0.021^*^	1.000
	Combination-30%	<0.001^*^	<0.001^*^	0.004^*^	1.000	0.114	0.469
Speed-30%	SM	<0.001^*^	<0.001^*^	<0.001^*^	0.974	0.003^*^	0.239
	Speed-20%	0.973	1.000	0.985	1.000	0.986	1.000
	Amplitude-20%	<0.001^*^	0.005^*^	0.002^*^	1.000	0.449	1.000
	Amplitude-30%	<0.001^*^	<0.001^*^	<0.001^*^	1.000	0.004^*^	1.000
	Combination-20%	<0.001^*^	0.003^*^	0.005^*^	1.000	0.055^(*)^	1.000
	Combination-30%	<0.001^*^	<0.001^*^	0.005^*^	1.000	0.153	0.996
Amplitude-20%	Standing measurement	<0.001^*^	<0.001^*^	<0.001^*^	0.674	0.003^*^	0.421
	Speed-20%	<0.001^*^	0.001^*^	0.001^*^	1.000	0.076	1.000
	Speed-30%	<0.001^*^	0.005^*^	0.002^*^	1.000	0.449	1.000
	Amplitude-30%	0.007^*^	0.297	0.092	1.000	0.384	1.000
	Combination-20%	1.000	1.000	1.000	0.997	0.627	1.000
	Combination-30%	0.005^*^	0.008^*^	0.057^(*)^	1.000	0.337	0.990
Amplitude-30%	Standing measurement	<0.001^*^	<0.001^*^	<0.001^*^	0.925	< 0.001^*^	0.540
	Speed-20%	<0.001^*^	<0.001^*^	<0.001^*^	1.000	0.001^*^	1.000
	Speed-30%	<0.001^*^	<0.001^*^	<0.001^*^	1.000	0.004^*^	1.000
	Amplitude-20%	0.007^*^	0.297	0.092	1.000	0.384	1.000
	Combination-20%	0.061^(*)^	0.968	0.643	1.000	1.000	1.000
	Combination-30%	0.821	0.100	0.755	1.000	0.799	0.838
Combination-20%	Standing measurement	<0.001^*^	<0.001^*^	<0.001^*^	1.000	0.004^*^	0.364
	Speed-20%	<0.001^*^	0.001^*^	0.002^*^	0.998	0.021^*^	1.000
	Speed-30%	<0.001^*^	0.003^*^	0.005^*^	1.000	0.055^(*)^	1.000
	Amplitude-20%	1.000	1.000	1.000	0.997	0.627	1.000
	Amplitude-30%	0.061^(*)^	0.968	0.643	1.000	1.000	1.000
	Combination-30%	0.011^*^	0.020^*^	0.121	1.000	0.911	0.888
Combination-30%	Standing measurement	<0.001^*^	<0.001^*^	0.001^*^	0.998	0.064	0.018^*^
	Speed-20%	<0.001^*^	<0.001^*^	0.004^*^	1.000	0.114	0.469
	Speed-30%	<0.001^*^	<0.001^*^	0.005^*^	1.000	0.153	0.996
	Amplitude-20%	0.005^*^	0.008^*^	0.057^(*)^	1.000	0.337	0.990
	Amplitude-30%	0.821	0.100	0.755	1.000	0.799	0.838
	Combination-20%	0.011^*^	0.020^*^	0.121	1.000	0.911	0.888

^*^Marks significant differences (*p* < 0.05) between the measurement conditions, (*) highlights comparisons just out of significance (*p* ≤ 0.065).

No significant differences were found in the COP parameters during conditions with the same amplitude (Speed-20% vs. Speed-30%, Amplitude-20% vs. Combination-20%, and Amplitude-30% vs. Combination-30%). Compared with Speed-20% and Speed-30%, all the remaining conditions resulted in a significant increase in COP-MedLat, COP-CranCaud, and support surface. A further increase in COP-MedLat was observed during Amplitude-30% compared to Amplitude-20% and Combination-20%, whereas no significant difference was found in the remaining parameters. The combination of the largest amplitude and fastest speed (Combination-30%) resulted in a significant increase in the COP-MedLat and COP-CranCaud (support surface compared to Amplitude-20%, just out of significance) compared with all other conditions.

Length in the function of surface was significantly increased during Combination-20% and Amplitude-30% compared to Speed-20%, and Amplitude-30% compared to Speed-30% (Combination-20% just out of significance). No significant differences were found between the statokinesiogram length and speed conditions.

The COP displacement was significantly larger in COP-CranCaud than COP-MedLat during standing measurement, Speed-20%, and Speed-30%, whereas no significant differences were observed during the remaining conditions (Table 5).

**Table 5:** Mean values, standard deviation, and *p*-values of COP-MedLat and COP-CranCaud during the same measurement condition.

Condition	COP-MedLat (mm)	COP-CranCaud (mm)	*p*-values
Standing measurement	5.94 ± 0.32^*^	12.55 ± 0.65^*^	<0.001
Speed-20%	23.23 ± 0.83^*^	30.26 ± 1.06^*^	<0.001
Speed-30%	25.42 ± 1.24^*^	32.24 ± 1.57^*^	0.002
Amplitude-20%	39.38 ± 1.73	43.85 ± 2.09	0.114
Amplitude-30%	51.24 ± 2.17	50.81 ± 1.67	0.877
Combination-20%	41.15 ± 2.13	46.22 ± 2.58	0.142
Combination-30%	58.77 ± 3.33	66.92 ± 4.24	0.151

^*^Significant difference between the COP-MedLat and COP-CranCaud groups.

## 4. Discussion

In the present study, three-dimensional circular movements were used to investigate the effects of external perturbations on the standing balance of sound dogs. We hypothesized that external perturbations challenge PS in dogs, which is reflected in the COP parameters of the body. Further, we hypothesized that higher amplitude and speed settings result in signs of increased instability and that an increase in amplitude has a stronger effect than an increase in speed. Thus, these hypotheses were partially confirmed.

With respect to the first hypothesis, it was possible to show that compared to standing measurement, each setting studied resulted in a significant increase in almost all COP parameters, indicating a challenge to PS. Furthermore, the amplitude setting has a greater effect than the speed setting. An increase in speed did not result in increased instability, because no significant difference was found in the COP parameters between the speed settings. This is partially consistent with what has been found in humans: Similar to our results, the speed of the perturbation on a motorized platform did not have a significant effect on COP-MedLat and COP-CranCaud in healthy individuals. However, statokinesiogram length significantly increased during the higher-speed setting (48). Therefore, future studies should use higher speeds than those used in this study to more precisely investigate this effect in dogs. While no comparable studies using a motorized platform have been performed in veterinary medicine, a similar pattern of results was obtained in blindfolded horses, leading to a significant increase in COP-MedLat and COP-CranCaud compared with measurements during undisturbed vision (23).

Based on the significant increase in COP parameters during settings with an increased amplitude, it can be concluded that, for future research with similar devices, settings with large amplitudes should be preferred over those with high speeds. While the combination of large amplitude and high speed resulted in the largest displacement in both axes, the setting used in this study (Combination-30%) was not well tolerated by the dogs.

Furthermore, the measurement results for condition Combination-30% showed a wide variation in the length in the function of surface. It can be assumed that the width of the confidence interval resulted in a lack of significance. The variation in the length in the function of surface values between dogs indicated that measurement condition Combination-30% was too challenging to obtain reliable data for the evaluation of COP parameters. This assumption is further corroborated by the fact that only nine of 13 dogs could stand still for the required measurement duration. Based on the descriptions of base of support in humans (34), it can be suggested that the COP exceeded the functional base of support in measurements in which the dogs did not stand still for the required time, which resulted in protective steps to prevent falling. Therefore, measurements using settings similar to Combination-30% are not recommended for future research projects.

During the standing measurement and speed conditions (Speed-20% and Speed-30%), the COP displacement was significantly larger in COP-CranCaud than in COP-MedLat. While the COP in humans shows a similar pattern with a larger migration in the craniocaudal direction, the results are contrary to the findings in ponies, which show a larger COP-MedLat than COP-CranCaud during the standing measurement of the forelimbs (15). Similarly, the support surface showed more pronounced migration in the mediolateral direction in dogs during standing measurement of the fore (11, 14) and hind limbs (11). While researchers have found that the fore and hind limbs can be used to determine the body COP in sound horses, the correlation coefficients between the total-body COP and forelimb data were higher than those of the hindlimb data. Therefore, the authors suggested preferring measurements of the forelimbs to assess the body COP in horses (31). In dogs, no data are available regarding the accuracy of these measurement procedures and conflicting outcomes should be viewed with caution because of different measurement techniques. Numerous anatomical differences between horses and dogs may explain the different compensatory mechanisms that maintain stability during standing measurement. Digitization in dogs may offer more stability in the mediolateral direction than that in unguligrade species (67). However, breed-specific compensation mechanisms during standing balance may be observed in dogs owing to differences in pressure distribution in the paws (68–70). Furthermore, the more versatile range of the shoulder and hip joints in dogs (67) may contribute to greater stability in the mediolateral direction. This is also supported by the fact that the mediolateral control of postural stability in humans relies mainly on a hip mechanism (71). The less pronounced abduction and adduction capabilities of the shoulder and hip joints in horses (67) could result in a limited compensatory mechanisms in the mediolateral axis during quiet standing.

More challenging conditions (Amplitude-20%, Amplitude-30%, Combination-20%, and Combination-30%) resulted in the lack of significant differences between COP-MedLat and COP-CranCaud. This can be explained by the rectangular shape of the base of support in dogs. As the mediolateral length of the base of support is smaller, the animals seem to be less stable in this direction, as proposed for ponies. Similarly, lame ponies showed a significantly larger displacement on the X-axis than that in the control group, whereas no difference in COP-CranCaud was observed (15). Therefore, maintaining an upright position on the mediolateral axis is more challenging than in the craniocaudal axis in horses and dogs. It has been proposed that larger forces are necessary to counteract the mediolateral disturbance of the PS, owing to the rectangular shape of the base of support observed in horses (23). This interpretation is in accordance with observations made during the tandem stance in humans. Similar to our results, a narrow base of support in the mediolateral axis compared to the normal standing position resulted in increased instability (53–55). However, a strong correlation between increased statokinesiogram length during sinusoidal external mechanical perturbations and COP-MedLat during a normal standing position has been previously described in human medicine; however, the authors did not include a comparison between COP-MedLat and COP-CranCaud (48). Furthermore, postural stability is challenged more during lateral than craniocaudal perturbations in healthy individuals and patients with Parkinson’s disease (49), which is in contrast to the theory that the increased length of the mediolateral axis of the base of support in humans provides better stability (72, 73). Therefore, it can be suggested that postural stability cannot be explained solely based on the shape of base of support, especially during external perturbations. In horses, it has been proposed that the extensor and flexor muscles are better developed than the abductor and adductor muscles and contribute to instability in the mediolateral direction (74), which could also be applied to dogs when PS is challenged.

Furthermore, human medical research has found a significant increase in COP displacement when an external perturbation is unpredictable compared to measurement procedures, where an anticipatory postural adjustment is possible owing to a predictable perturbation. These results lead to the conclusion that postural control training plans are valuable for improving the interaction between anticipatory and compensatory PS (75). Similarly, repetitive measurements using predictive forward and backward translation on a motorized platform led to a significant decrease in COP displacement. In conclusion, the human body adapts to predictive and repetitive perturbations by modifying muscle activity (46). Given the sinusoidal movements of the selected settings, the external perturbation used in this study can be classified as predictable. Therefore, future studies using both predictable and unpredictable settings may provide further insights into whether dogs respond similarly to humans when unpredictable external perturbations are applied to an animal’s body. These measurements should be supplemented by the analysis of electromyographical activity to investigate prior muscle activation when a perturbation is predictable, as described in human medicine (46, 75). Muscle activation and COP displacements differed between elderly and young individuals (75), and, as previously mentioned, senile (>75% of expected lifespan) (16) and orthopedically diseased dogs (11, 14) showed alterations in COP parameters compared to young and sound dogs during standing measurement. Therefore, alterations in muscle activation strategies in senile and orthopedically diseased dogs should be the focus of future research.

Balancing exercises using wobble boards and cushions are an integral part of the rehabilitation of neurological and orthopedic diseases to improve muscular function and PS (76, 77). Furthermore, a training program that includes PS challenges is recommended in sports dogs to prevent injury risk (77, 78) and in elderly animals to maintain a good quality of life (60). However, the assumption that proprioceptive training programs positively affect PS is based on human medical studies (79). Human research has found that balance recovery effectiveness can be trained or relearned, and perturbation-based exercises have shown positive impacts on reactive balance performance in post-stroke (80, 81), Parkinson’s disease (42), and patients with anterior cruciate ligament rupture (7, 8). Therefore, future veterinary medicine studies should focus on the effects of PS training programs on diseased animals.

The investigated settings can be used as an orientation for the implementation of external perturbations in rehabilitation and research on diseases that are considered to negatively impact PS. The following graduated scheme can be suggested: because speed is less challenging for PS than the amplitude, weak dogs should start with a small amplitude (Speed-20%, Speed-30%). This difficulty can be increased by increasing the amplitude (Amplitude-20%, Combination-20%); however, it should be considered that Combination-20% does not have superior effects to Amplitude-20%. The combination of high speed and large amplitude (Combination-30%) further increases the challenge in the mediolateral and craniocaudal directions. However, sound dogs do not perform well under these conditions. Therefore, a further increase should first be performed using a larger amplitude with a slow speed (Amplitude-30%), which is advantageous in the mediolateral direction.

One limitation of our study was the small sample size of 13 dogs. Even though the inclusion criteria were designed to limit this study to healthy, adult dogs with similar body type and weight, it should be mentioned that previous research found a significant correlation between weight, height, and length and the outcomes of COP measurements (16).

## 5. Conclusion

To the best of our knowledge, this is the first study to demonstrate that external mechanical perturbations challenge PS in dogs. All conditions led to a significant increase in COP-MedLat, COP-CranCaud, and support surface compared with standing measurement. In addition, the extent of displacement positively correlated with the increase in amplitude. All the tested settings, except for Combination-30%, which was not well tolerated by the dogs, are recommended for future research and training programs. While the COP displacement was significantly larger in the craniocaudal direction than that in the mediolateral direction during standing measurement and settings with a low amplitude, no significant difference was found during the more challenging conditions. Therefore, it can be suggested that dogs are less stable during a mediolateral disturbance of PS. Further studies should address the effects of external perturbations on COP parameters in juvenile, senile, and orthopedically or neurologically diseased dogs. These measurements should be supplemented with the determination of muscular activity using electromyographic analysis.

## Data availability statement

The raw data supporting the conclusions of this article will be made available by the authors, without undue reservation.

## Ethics statement

The animal studies were approved by Ethics and Animal Welfare Committee of the University of Veterinary Medicine, Vienna. The studies were conducted in accordance with the local legislation and institutional requirements. Written informed consent was obtained from the owners for the participation of their animals in this study.

## Author contributions

BB, CP, and CL: conceptualization, methodology, and validation. AT: formal analysis. CL and RG: data curation. CL: writing—original draft preparation. BB, MM, CP, BR, and CL: writing—review and editing. All authors contributed to the article and approved the submitted version.

## Funding

Allcare Innovations (111 Rue Carl von Linné, 26500 Bourg les Valence, France) reduced the purchase cost of the Imoove® Vet by 40% to ensure that the study was financially viable.

## Conflict of interest

The authors declare that the research was conducted in the absence of any commercial or financial relationships that could be construed as a potential conflict of interest.

## Publisher’s note

All claims expressed in this article are solely those of the authors and do not necessarily represent those of their affiliated organizations, or those of the publisher, the editors and the reviewers. Any product that may be evaluated in this article, or claim that may be made by its manufacturer, is not guaranteed or endorsed by the publisher.
