# Corrigendum: External mechanical perturbations challenge postural stability in dogs

**DOI:** 10.3389/fvets.2025.1630608

**Published:** 2025-06-10

**Authors:** Christiane Lutonsky, Christian Peham, Marion Mucha, Bianca Reicher, Rita Gaspar, Alexander Tichy, Barbara Bockstahler

**Affiliations:** ^1^Department of Companion Animals and Horses, University Clinic for Small Animals, Small Animal Surgery, Section of Physical Therapy, University of Veterinary Medicine, Vienna, Austria; ^2^Department of Companion Animals and Horses, University Clinic for Horses, Movement Science Group, University of Veterinary Medicine, Vienna, Austria; ^3^Department of Biomedical Sciences, Bioinformatics and Biostatistics Platform, University of Veterinary Medicine, Vienna, Austria

**Keywords:** canine balance, center of pressure, postural stability, posturography, veterinary rehabilitation, external mechanical perturbations

In the published article, there was an error in form of a distortion of words concerning the results of one comparison (COP-MedLat vs. COP-CranCaud). While the tables (Table 4 and Table 5), discussion and conclusion offer the right findings, the abstract and conclusion state the opposing.

A correction has been made to **Abstract**, page 1.

This sentence previously stated:

“The mediolateral COP displacement was significantly greater than the craniocaudal COP displacement during standing measurement and conditions with a small amplitude, whereas no significant difference was observed during settings with an increased amplitude.”

The corrected sentence appears below:

“The craniocaudal COP displacement was significantly greater than the mediolateral COP displacement during standing measurement and conditions with a small amplitude, whereas no significant difference was observed during settings with an increased amplitude.”

A correction has been made to **Results**, *3.3 Center of pressure*, page 6, paragraph 6.

This sentence previously stated:

“The COP displacement was significantly larger in COP-MedLat than in COP-CranCaud during standing measurement, Speed-20%, and Speed-30%, whereas no significant differences were observed during the remaining conditions (Table 5).”

The corrected sentence appears below:

“The COP displacement was significantly larger in COP-CranCaud than COP-MedLat during standing measurement, Speed-20%, and Speed-30%, whereas no significant differences were observed during the remaining conditions (Table 5).”

The authors apologize for this error and state that this does not change the scientific conclusions of the article in any way. The original article has been updated.

